# Comparison of the sympathetic stimulatory abilities of B-type procyanidins based on induction of uncoupling protein-1 in brown adipose tissue (BAT) and increased plasma catecholamine (CA) in mice

**DOI:** 10.1371/journal.pone.0201203

**Published:** 2018-07-30

**Authors:** Yuta Nakagawa, Kana Ishimura, Satomi Oya, Masaki Kamino, Yasuyuki Fujii, Fumio Nanba, Toshiya Toda, Takeshi Ishii, Takahiro Adachi, Yoshitomo Suhara, Naomi Osakabe

**Affiliations:** 1 Department of Bio-science and Engineering, Shibaura Institute of Technology, Munumaku, Saitama, Japan; 2 Department of Research and Development, Fujicco. Co. Ltd., Kobe, Hyogo, Japan; 3 Department of Nutrition, Kobe Gakuin University, Kobe, Japan; 4 Department of Immunology, Medical Research Institute, Tokyo Medical and Dental University, Tokyo, Japan; Medical University of Vienna, AUSTRIA

## Abstract

**Objectives:**

We previously found that elevated energy expenditure following a single oral dose of flavan 3-ols (FL), a mixture of catechins and B type procyanidins, is caused by sympathetic nerve activation. In the present study, we compared the activity of the FL components (-)-epicatechin (EC; monomer), procyanidin B2 (B2; dimer), procyanidin C1 (C1; trimer), cinnamtannin A2 (A2; tetramer), and more than pentamer fraction (P5).

**Methods:**

Male ICR mice were treated with a single oral dose of FL, EC, B2, C1, A2, or P5. The animals were sacrificed and blood and brown adipose tissue (BAT) sampled. The plasma catecholamine (CA) levels and BAT uncoupling protein (UCP)-1 mRNA expression were determined.

**Results:**

A single dose of 10 mg/kg FL significantly increased plasma CA and UCP-1 mRNA levels. B2, C1, and A2, but not EC and P5 (all at 1 mg/kg), significantly increased plasma adrenaline levels. Plasma noradrenaline was significantly elevated by B2 and A2, but not by EC, C1, or P5. UCP-1 mRNA levels were significantly increased by C1 and P5. In the dose response study of A2, 10^−3^ mg/kg A2 increased UCP-1 mRNA levels significantly, but not 10^−2^ and 10^−1^ mg/kg A2. In addition, combination treatment with 10^−1^ mg/kg A2 and yohimbine, an α2 adrenalin blocker, remarkably increased UCP-1 mRNA levels.

**Conclusion:**

These results suggest that FL and its components, except EC, increase UCP-1 mRNA and plasma CA with varying efficacy.

## Introduction

Flavan 3-ols (FL), as a mixture of monomeric catechins and B type oligomer procyanidins, are enriched in several plant foods, including cocoa beans [1], red wine [2], and black soy beans [3]. Foods rich in FL could have significant potential for managing cardiovascular health [4–6]. Monomeric catechins are more readily absorbed from the gastrointestinal tract and almost all are metabolized; therefore, unchanged forms are nearly absent in blood [7]. Procyanidins are rarely absorbed from the gut into the blood [8–10]. A recent comprehensive review of polyphenols and human cardiovascular health suggested that the mechanism underlying the beneficial effect of FL has not been fully elucidated because of their poor bioavailability [11]. Consequently, focus has been placed on the metabolome analyses of feces, which may provide insight into the chronic physiological changes associated with the repeated ingestion of FL [12].

Despite poor bioavailability, FL exert postprandial actions on the hemodynamic [13], metabolic [14], and nervous systems [15] a few hours after ingestion. Thus, the actions seem to be unrelated to metabolites when the passage time in the intestine is taken into account [16]. In addition, a significant increase in energy expenditure, uncouple protein (UCP)-1 mRNA expression in brown adipose tissue (BAT), and plasma adrenaline (AD) were observed a few hours after FL ingestion in our previous animal study. However, (-)-epicatechin (EC), a monomeric FL component, did not show these alterations [17]. These results suggested that there is a difference in the metabolic alteration between catechins and procyanidins.

**Fig 1:**
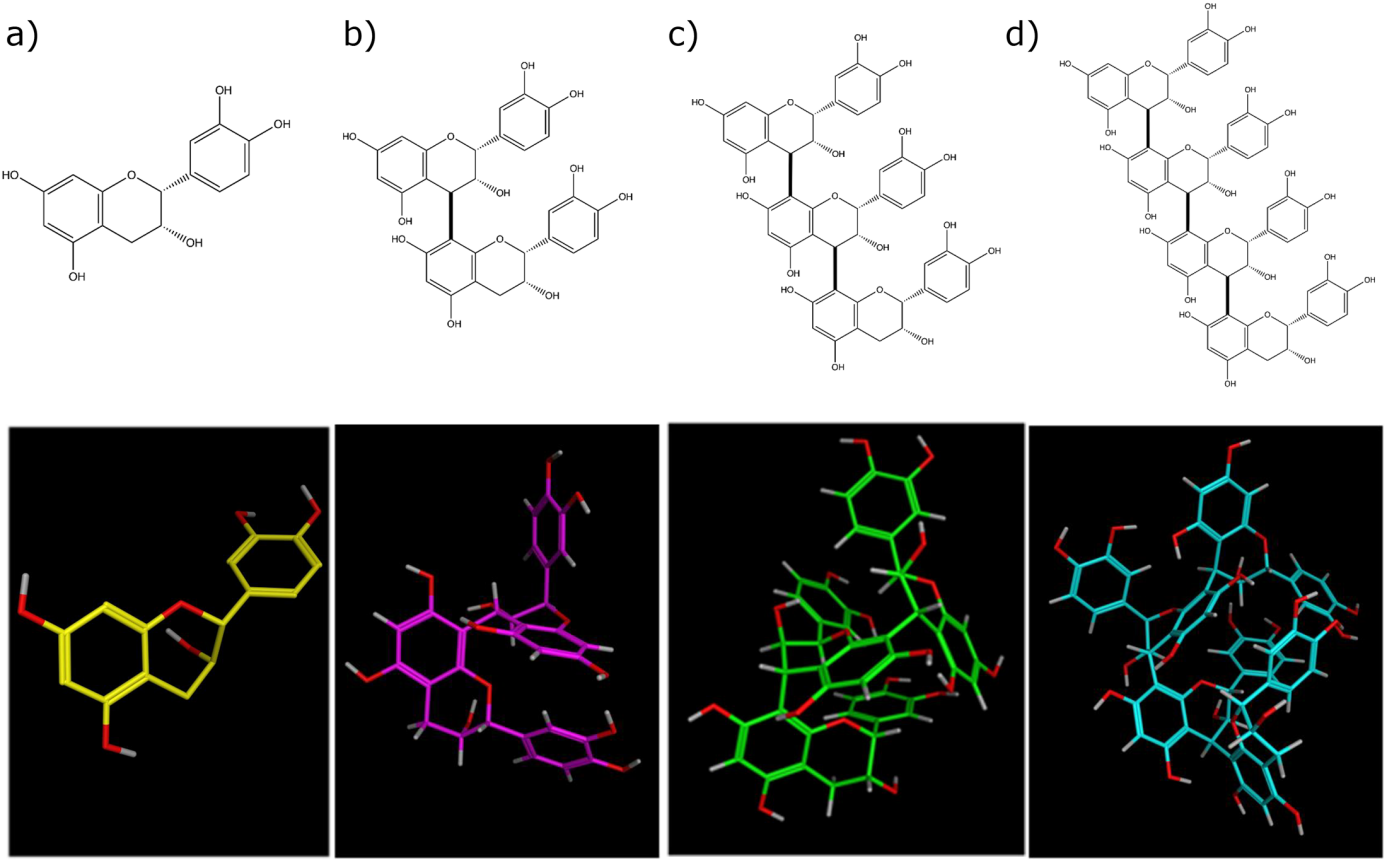
Structures of B type procyanidin. (a) (-)-epicatechin (EC; monomer), (b) procyanidin B2 (B2; dimer), (c) procyanidin C1 (C1; trimer), (d) cinnamtannin A2 (A2; tetramer).

In the present study, we compared the efficacy of FL components (-)-EC (monomer), procyanidin B2 (B2; dimer), procyanidin C1 (C1; trimer), cinnamtannin A2 (A2; tetramer), and more than pentamer fraction (P5) (Fig 1) on the postprandial metabolic action by determining plasma catecholamine (CA) concentrations and BAT UCP-1 mRNA levels.

## Materials and methods

### Materials

FL from black soybean seed coats were prepared according to Ito et al. [3] (S1 Methods). The FL extraction contained 6.22% catechins, 6.35% B2, 2.69% C1, and 1.25% A2. As a reference, we also determined the polyphenol concentration in this fraction using the vanillin-sulfuric acid method (77.10%). Procyanidins were further purified by preparative reversed phase HPLC to ≥95%, except P5 (S1 Table).

### Animals and diets

The study was approved by the Animal Care and Use Committee of Shibaura Institute of Technology (Permit Number: 27–2956). All mice received humane care under the guidelines of this institution. Male ICR mice weighing 35–40 g were obtained from Charles River Laboratories Japan, Inc. (Tokyo, Japan). Four mice were placed in a cage and kept in a temperature-regulated room (23–25°C) with controlled lighting (12/12 h light/dark cycles) and freely accessed water and diet. Basal diet (MF^®^) was obtained from Oriental Yeast Co, Ltd. (Tokyo, Japan), the composition of which is provided in the Supplemental Information (S2 Table). In order to avoid suffering and an influence of anesthesia on blood CA dynamics, all animals were sacrificed by decapitation by skilled researchers according to the experimental procedure.

#### Experiment 1

After 4 days on a basal diet, 8 to 10-week-old mice were divided into two experimental groups. The vehicle group was given distilled water (vehicle), and the FL group was given 10 mg/kg body weight FL by oral gavage. Ten milligrams per kilogram FL was equivalent to 25–40 g dark chocolate per human, when converted from the previous reports regarding with FL content in commercially available dark chocolate [18–20]; we previously confirmed an increase in sympathetic nervous activity in mice [17]. Before treatment and 1, 2, or 4 h after treatment (n = 8 each), the animals were decapitated and blood collected in ethylenediaminetetraacetic acid (EDTA). After cutting with scissors approximately 1–2 cm below the neck at the back of the mouse, we took the brown adipose tissue (BAT), which was a triangular shape under the white fat [21]. Tissue samples were collected by dissection, snap frozen in liquid nitrogen, and stored at -80°C until analysis.

#### Experiment 2

Forty-eight mice were divided into six groups. The vehicle group was treated with distilled water and the mice in the EC, B2, C1, A2, and P5 groups were treated with 1 mg/kg of the FL component under investigation in the group. EC was obtained from Tokyo Chemical Industry (Tokyo, Japan) and the other procyanidins were obtained by the method shown in supplemental information (S1 Methods). The dose was determined based on our previous study comparing the sympathetic stimulation effect of various polyphenols [22]. Two hours after treatment, the animals were decapitated and blood and BAT collected as described above.

#### Experiment 3

Fifty-six mice were divided into seven groups. The vehicle group (n = 8) was given distilled water orally. Four groups were treated with vehicle, 10^−3^, 10^−2^, or 10^−1^ mg/kg A2. The other two groups were given an intraperitoneal injection of 0.25 mg/kg yohimbine (YO), an α2 adrenaline receptor (AR) blocker, according to the method of our previous report [23]. Ten minutes later, one group of yohimbine-treated mice (n = 8) were given distilled water orally (yohimbine-vehicle group), and the other 8 mice were given 10^−1^ mg/kg A2(yohimbine-10^−1^ mg/kg A2 group). Two hours after treatment, the animals were decapitated and BAT collected as described above.

#### HPLC analysis of plasma catecholamine concentrations

Plasma CA were analyzed by HPLC-electrochemical detection after being prepared using a monolithic silica disk-packed spin column (MonoSpin, GL Science, Tokyo Japan) [24]. Norepinephrine and epinephrine were obtained from Tokyo Kasei (Tokyo, Japan). Dopamine was acquired from Wako Pure Chemical (Tokyo, Japan). The 3,4-dihydroxybenzylamine was from Sigma Aldrich (Japan). Acetonitrile was purchased from Wako Pure Chemical (Tokyo, Japan). Plasma, 1 M phosphate buffer (pH 8.0; 50 μL), and 400 ng/mL DHBA (internal standard; 40 μL) were directly injected into the pre-activated spin column and centrifuged at 3000 rpm for 5 min. The column was then rinsed with 200 μL of 100 mM phosphate buffer (pH 8.0) by centrifugation. Finally, the column was installed into a new microtube, and the analytes adsorbed onto the column were eluted with 1% acetic acid (200 μL). A 20-μL aliquot of the eluate was injected into the HPLC system (Prominance HPLC System Shimazu Corporation, Kyoto Japan) equipped with an ECD (ECD 700 S, Eicom Corporation, Kyoto Japan) set at 650 mV. HPLC separation was performed on an Inertsil ODS-4 (250×3.0 mm I.D., 5 μm; GL Science) at 35°C and a flow rate of 0.5 mL/min using a mobile phase comprised of 20 mM sodium acetate-citrate buffer/acetonitrile (100/16, v/v) containing 1 g/L sodium 1-octanesulfonate.

#### Quantitative RT-PCR analysis

Total RNA was prepared from BAT using TRIzol reagent (Life Technologies, California, USA) according to the manufacturer’s instructions. Briefly, 10 μg total RNA was reverse-transcribed in a 20 μL reaction volume with high capacity cDNA Reverse Transcription kits (Life Technologies, California, USA). Real-time reverse-transcription PCR was performed using 100 ng total cDNA in the StepOne^™^ Real-Time PCR System (Life Technologies, California, USA). Primer and probe sequences were selected using a Taqman^™^ Gene Expression Assay (Life Technologies, California, USA) and the following genes: GAPDH (Mm99999915_g1) and UCP-1 (Mm_01244861_m1) from Life Technologies, Glyceraldehyde-3-phosphate dehydrogenase (GAPDH) was used as an internal control. The buffer used in the systems was THUNDER BIRD Prove qPCR Mix (TOYOBO, Tokyo, Japan). The PCR cycling conditions were 95°C for 1 min, followed by 40 cycles at 95°C for 15 s and 60°C for 1 min.

#### Data analysis and statistical methods

The data were expressed as means and standard deviations. Statistical analyses were performed using one or two-way ANOVA and post hoc comparisons and the Tukey-Kramer method. P<0.05 was considered significant.

## Results

### Experiment 1

**Fig 2:**
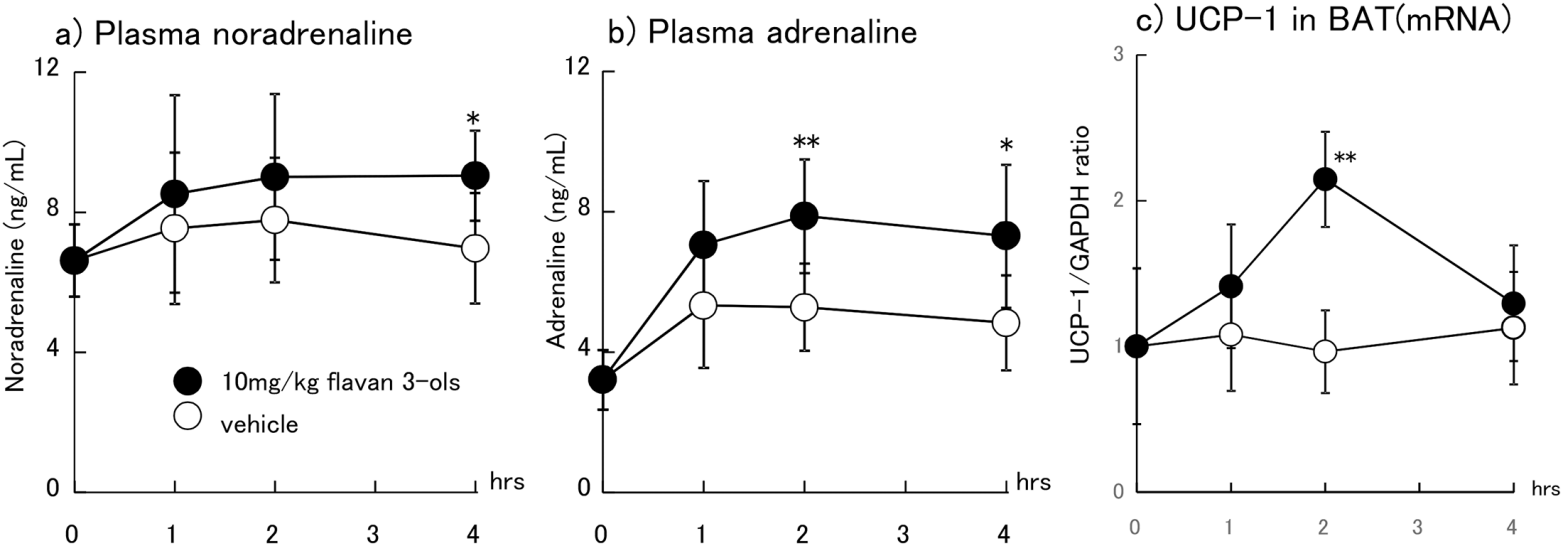
Alterations in plasma noradrenaline (a), adrenaline (b), and UCP-1 mRNA levels in BAT (c) after ingestion of 10 mg/kg flavan 3-ols or vehicle. The values represent mean ± standard deviation (each group, n = 8). *p<0.05, **p<0.01 (Tukey-Kramer test vs. vehicle).

The alteration of plasma CA concentrations and UCP-1 mRNA expression following ingestion of 10 mg/kg FL is shown in Fig 2. Plasma NA levels were elevated significantly 4 h after ingestion of FL (p = 0.022) compared to vehicle treatment (Fig 2a). The plasma AD levels were also increased significantly 2 and 4 h after administration of FL (p = 0.005 and 0.018, respectively; Fig 2b). UCP-1 mRNA levels were significantly increased 2 h after ingestion of FL (p = 0.001; Fig 2c).

### Experiment 2

**Fig 3:**
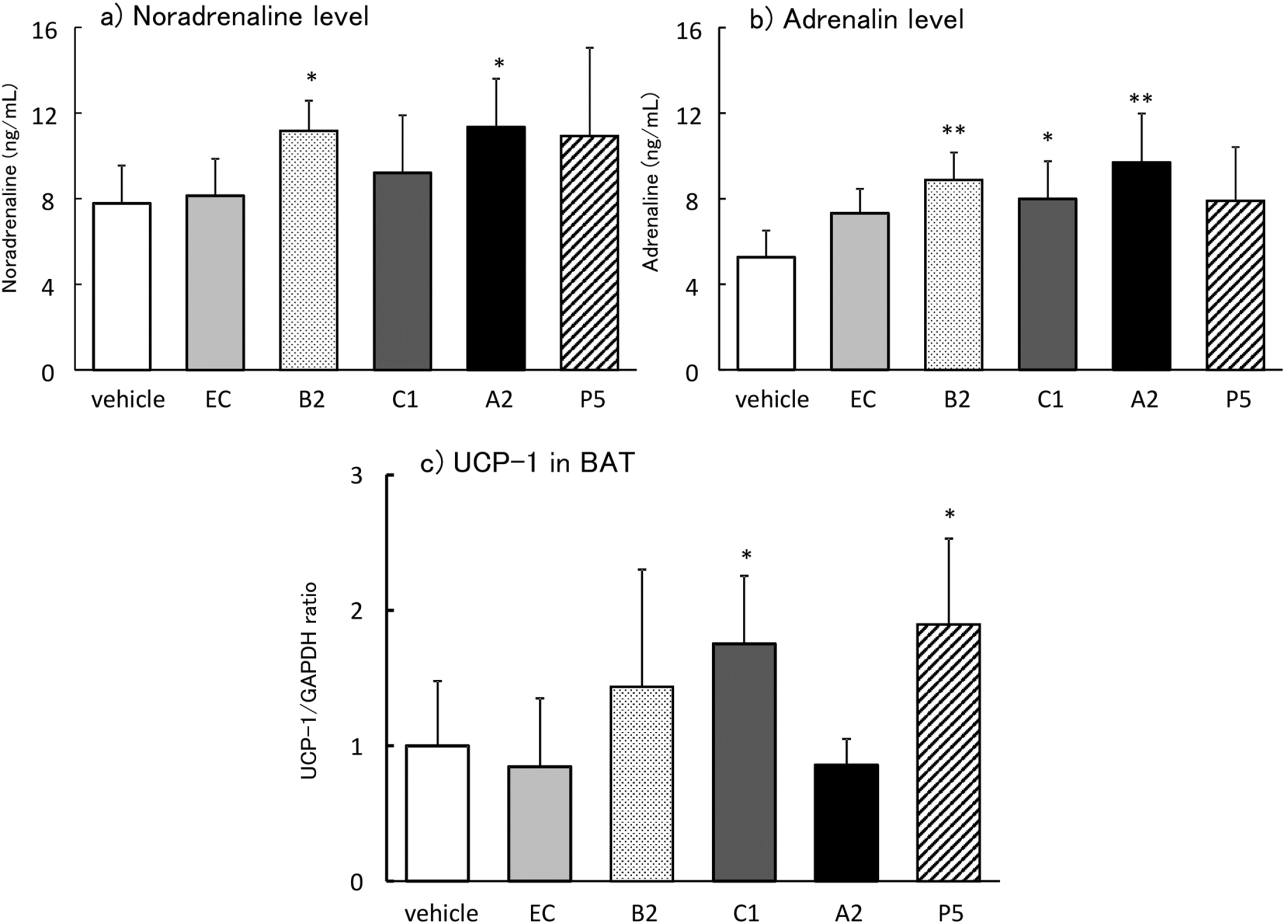
Alterations in plasma noradrenaline (a), adrenaline (b), and UCP-1 mRNA in BAT (c) 2 h after ingestion of 1 mg/kg EC, B2, C1, A2, P5, or vehicle. The values represent mean ± standard deviation (each group, n = 8). **p<0.01. *p<0.05, **p<0.01 (Tukey-Kramer test vs. vehicle)

In experiment 1, plasma AD and UCP-1 mRNA alterations peaked 2 h after ingestion. Therefore, we compared the alterations 2 h after treatment with 1 mg/kg EC, B2, C1, A2, and P5. Plasma NA levels significantly increased only in the B2 and A2 groups (p = 0.034 and 0.049, respectively) compared to the vehicle group (Fig 3a). This elevation was not observed in the EC, C1, or P5 groups. Plasma AD levels were significantly increased after treatment with B2, C1, or A2 (p = 0.003, 0.041, or 0.0002, respectively; Fig 3b). In contrast, UCP-1 mRNA levels in BAT significantly increased only in the C1 and P5 groups (P = 0.046 and 0.041, respectively) compared to the vehicle group (Fig 3c), and not the EC, B2, or A2 groups.

### Experiment 3

**Fig 4:**
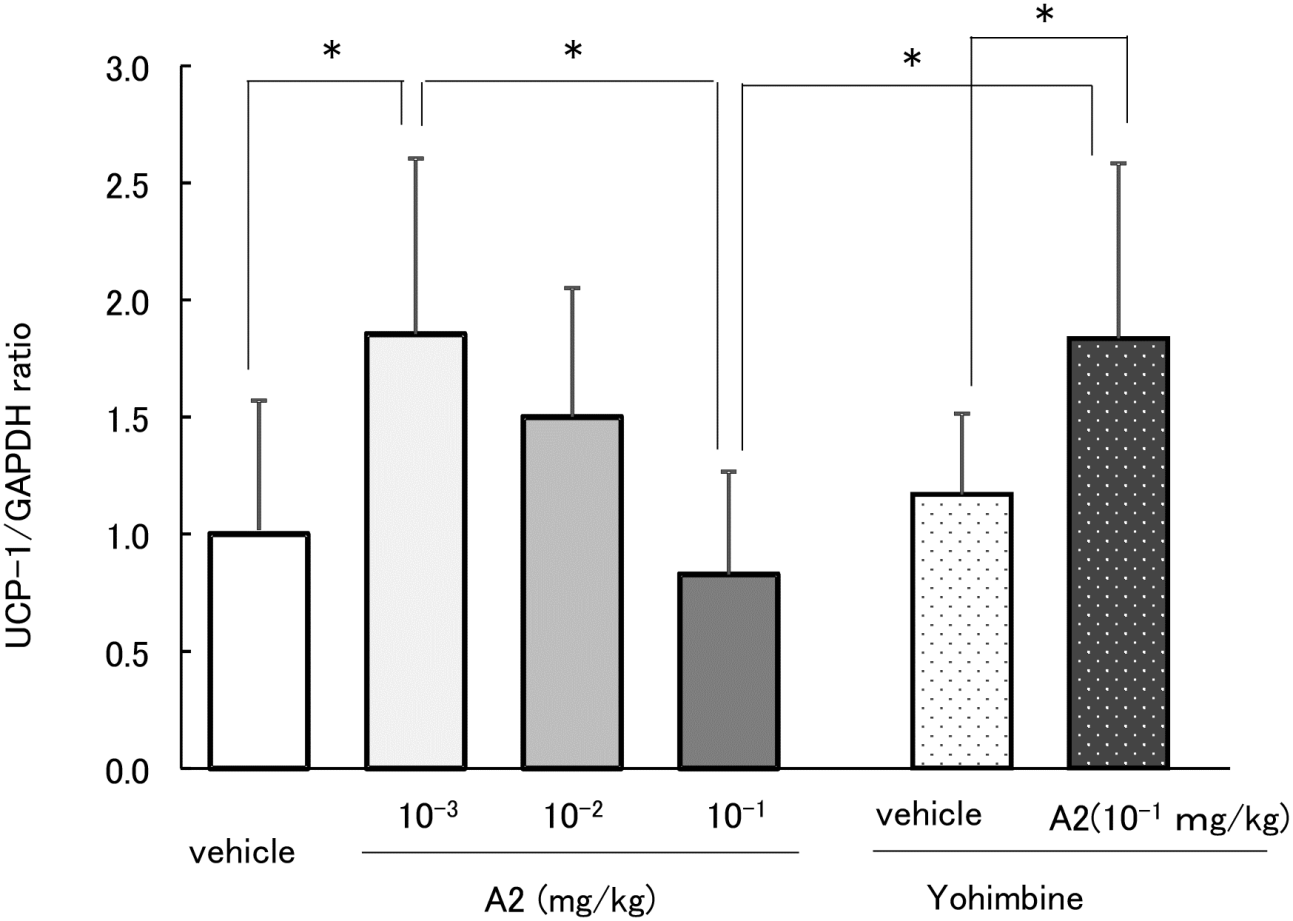
Dose-reactive response of UCP-1 mRNA levels to A2 and A2 plus yohimbine in BAT. The values represent mean ± standard deviation (each group, n = 8). *p<0.05, **p<0.01 (Tukey-Kramer test between experimental group).

We evaluated the dose reactive activity of A2 and the interaction between high dose of A2 and α2 AR blocker, in order to elucidate inconsistent results between rising plasma CA concentration and UCP-1 mRNA level as shown in experiment 2. In our previous report, FL did not exhibit sympathetic hyperactivity at high doses [23]; therefore, the dosage of A2 was gradually reduced to 10^−1^, 10^−2^, and 10^−3^ mg/kg. As shown in Fig 4, UCP-1 mRNA levels significantly increased in the 10^−3^ mg/kg A2 group (p = 0.021), but this change was not observed in the 10^−2^ and 10^−1^ mg/kg groups. In contrast, combination treatment with 10^−1^ mg/kg A2 and yohimbine increased UCP-1 mRNA levels in BAT (Fig 4). A significant difference was found between 10^−3^ mg/kg A2 with or without yohimbine (p = 0.025).

## Discussion

In the present study, plasma AD was increased 2 and 4 h and NA 4 h after ingestion of 10 mg/kg FL. Plasma CA, mainly as AD and marginal NA, are secreted from the adrenal medulla into the bloodstream after sympathetic nerve activation [25, 26]. Plasma CA have been reported to enhance the energy metabolism of the whole body, including skeletal muscle and white adipose tissue. In addition, metabolic change in skeletal muscle and white adipocytes has been shown to be enhanced by rising plasma CA [27–30]. Elevation of blood CA was considered one of the reasons for increased energy metabolism after a single administration of FL in our previous study [17].

CA are also secreted from the sympathetic end to an effector organ as a neurotransmitter [31]. β3AR is one of the sympathetic target receptors expressed in BAT. NA released from the sympathetic nerve activates β3AR on BAT, leading to thermogenic effects [32, 33]. Transient adrenergic stimulation, such as cold exposure, increases cytosolic free fatty acid levels essential for mitochondrial energy dissipation, along with upregulation of UCP-1 mRNA [34]. As in our previous study, up-regulation of UCP-1 mRNA 2 h after a single oral dose of FL was considered to be induced by enhanced sympathetic nerve activity [17, 35].

FL derived from black soy bean coats include more than 30mer catechins detected by MALDI-TOFMS [36], and most part of them are less than tetramers [3]. Several reports have compared the antioxidative activities of FL constituents *in vitro* [37–39], but *in vivo* studies are sparse. In the present study, we compared sympathetic nerve stimulation based on the increase of UCP-1 mRNA expression in BAT and increased plasma CA levels. The peak plasma AD and UCP-1 mRNA alterations were 2 h after ingestion of FL. Therefore, we compared these alterations between component groups 2 h after administration. The results suggest that B2 and A2 were more potent than EC or C1 in elevating plasma CA. In contrast, UCP-1 mRNA levels significantly increased in the C1 and P5 groups, but not the EC, B2, or A2 groups (Fig 3c). Inconsistent results were found between plasma CA elevation and UCP-1 mRNA upregulation.

We previously found similar paradoxical results in hemodynamics. From 1 to 10 mg/kg FL increased heart rate and blood pressure transiently, but these changes were not observed with 100 mg/kg FL [23], and the combination treatment of 100 mg/kg FL and α2 AR blocker yohimbine increased heart rate and blood pressure to a greater extent. Yohimbine is known to block α2AR, an inhibitory receptor mainly distributed in the central nervous system, including the vasomotor center [40]. High FL dose was suggested to promote excessive stimulation of the dominant noradrenergic nerve, with subsequent activation of inhibitory α2AR in the vasomotor center, resulting in inhibition of CA release from the sympathetic nerve. A recent report also suggested that the premotor neurons controlling thermogenic effector activation are primarily within the region of the medullary rostral raphe pallidus (rRPa) [41]. Non-shivering and shivering thermogenesis are inhibited by α2AR activation in the rRPa [42]. We previously examined whether excessive A2 activated α2AR in rRPa using yohimbine [43] after a dose response test of A2. Administration of 10^−2^ to 1 mg/kg A2 did not elevate UCP-1 mRNA expression, but UCP-1 was upregulated significantly 2 h after ingestion of 10^−3^ mg/kg A2 (Figs 3 and 4). In addition, combination treatment with yohimbine and 10^−1^ mg/kg A2, which did not cause any change alone, showed significant elevation of UCP-1 mRNA. High dose A2 was suggested to activate α2AR in rRPa. Consequently, the release of CA from sympathetic nerve endings was reduced and β3 receptor was not activated.

Oligomers had a greater ability than monomer in up-regulating UCP-1 mRNA. The present results confirmed previous findings that the fraction of procyanidin oligomers was more reduced in the presence of obesity complications induced by high fat diet than with the monomer or polymer fraction [44].

We previously reported that repeated FL dose promoted mitochondrial biogenesis [42], but further study was needed to elucidate each procyanidin in mitochondrial respiration using high-resolution Oxygraph-2k or the sensitive high-throughput Seahorse XF Extracellular Flux Analyzer. In the present study, A2 showed potent activity on sympathetic nerve stimulation, whereas similar but weak activity was detected with B2 or C1, but not EC, treatment. We administered the same amounts of catechins and procyanidins to the animals; therefore, the number of hydroxyl groups from procyanidins were not important to the sympathetic nerve activation. B type procyanidins may have a common core structure, which is indispensable for interacting with a target biomolecule. Polymerized 2-phenyl-3,4-dihydro-2H-chromen-3-ol is a possible core structure (Fig 1), and A2 has been suggested to have a complementary relationship with the target biomolecule that is not yet known.

EC has been reported to be absorbed via the gastrointestinal tract, but procyanidin oligomers were present in the blood at very low concentrations [7–10]. Despite poor bioavailability, procyanidin oligomers, especially A2, exhibited greater UCP-1 up-regulation, and these changes were followed soon after (2 h) a single dose of procyanidin, i.e., it seems to be unrelated to their metabolites from the perspective of passage time in the intestine. These effects were suggested to be due to enhanced sympathetic nerve activity, but there were great differences in the activity between procyanidins. These results suggest that specific target molecules recognizing procyanidins exist in the upper gastrointestinal tract.

## Conclusions

In conclusion, FL and the mixture’s components, except EC, increase plasma CA and BAT UCP-1 mRNA levels via sympathetic nerve stimulation, but with varying efficacy. Because the activity did not correlate with the number of hydrogen groups, the differences may be due to the stereochemical structure. Further studies are needed regarding the structure-activity correlation between B type procyanidins or other polyphenols on the sympathetic nerve stimulation activity in order to predict the target biomolecule of polyphenol.

## Supporting information

S1 Methods Extraction of flavan 3-ol fraction and each procyanidin from black soybean seed coat. (PDF)

S1 Table Recovered amount from 100g of seed coat and purity of each chemical. (PDF)

S2 Table Component of basal diet(MF^®^, in 100g). (PDF)
