# Supplementary material for: Comparison of the sympathetic stimulatory abilities of B-type procyanidins based on induction of uncoupling protein-1 in brown adipose tissue (BAT) and increased plasma catecholamine (CA) in mice
Source: PLoS One. 2018 Jul 30;13(7):e0201203. doi: 10.1371/journal.pone.0201203 (PMC6066223; doi:10.1371/journal.pone.0201203)
Supplement: S1 Methods — (PDF) [file pone.0201203.s001.pdf]

S1 Methods

Extraction of flavan 3-ol fraction and each procyanidin from black soybean seed coats

Black soybean seed coats (100 g) were subjected to extraction with a 70% acetone/0.5% acetic acid solution (2000 mL) for 3 h at room temperature. The extract was concentrated by rotary evaporation in a 20°C water bath. After filtration, the concentrated extract (approximately 6.2 g solid in 200 mL) was applied to a 100 mL Sepabeads SP700 column (Mitsubishi Chemical Corp., Japan). After rinsing the column with distilled water, the fraction containing procyanidins was eluted with 60% EtOH. This eluation fraction was concentrated by rotary evaporation in a 20°C water bath and freeze-dried to obtain the flavan 3-ol (FL) fraction. Each procyanidin was isolated from the FL fraction by high-performance liquid chromatography (HPLC) [1]. Procyanidin B2 (B2) and C1 (C1) were obtained as follows. The FL fraction (200 µL) was injected onto a Unison UK-C18 column (3 µm, φ3×150 mm, 35°C; Imtakt Corporation, Kyoto, Japan) and eluted with a mixture of (A) 0.1% formic acid in water and (B) acetonitrile as the mobile phase. Separations were performed using 5–50% linear gradients of B in A (0–45 min) at a flow rate of 5.0 mL/min and detected at 280 nm.

Cinnamtannin A2 (A2) was obtained as follows. The FL fraction (200 µL) was injected onto a Unison UK-C18 column (5 µm, φ20×250 mm, 35°C) and eluted with a mixture of (A) 0.1% formic acid in water and (B) acetonitrile as the mobile phase. Separations were performed using 5–25% linear gradients of B in A (0–135 min) at a flow rate of 5.0 mL/min and detected at 280 nm. The P5 fraction was obtained by excluding the peak of B2, C1, and A2 in the same procedure as A2. The purity of B2, CA, and A2 and concentration of them in the FL fraction were evaluated using the already obtained pure compound as a standard product [1]. Briefly, each fraction (5 µL) was injected onto a Unison UK-C18 column (3 µm, φ3×150 mm, 35°C) and eluted with a mixture of (A) 0.1% formic acid in water and (B) acetonitrile as the mobile phase. Separations were performed using 5–50% linear gradients of B in A (0–45 min) at a flow rate of 5.0 mL/min and detected at 280 nm. The amount recovered from 100 g of seed coat and purity of each chemical is provided in S1 Table.

Reference

1. Ito C, Oki T, Yoshida T, Nanba F, Yamada K, Toda T. Characterisation of proanthocyanidins from black soybeans: isolation and characterisation of proanthocyanidin

oligomers from black soybean seed coats. Food Chem. 2013;141(3):2507-12. Epub 2013/07/23. doi: 10.1016/j.foodchem.2013.05.039. PubMed PMID: 23870988.
