# Supplementary material for: Comparison of the sympathetic stimulatory abilities of B-type procyanidins based on induction of uncoupling protein-1 in brown adipose tissue (BAT) and increased plasma catecholamine (CA) in mice
Source: PLoS One. 2018 Jul 30;13(7):e0201203. doi: 10.1371/journal.pone.0201203 (PMC6066223; doi:10.1371/journal.pone.0201203)
Supplement: S1 Table — (PDF) [file pone.0201203.s002.pdf]

S1 table Recovered amount from 100 g of seed coat and purity of each chemicals

	amount(mg)	purity (%)†
Procyanidin B2	131	98.7
Procyanidin C1	41.7	97.1
Cinnamtannin A2	7.5	97.6
P5 fraction	326.8	−

The value showed the average of duplicate measurements
