# Supplementary material for: Comparison of the sympathetic stimulatory abilities of B-type procyanidins based on induction of uncoupling protein-1 in brown adipose tissue (BAT) and increased plasma catecholamine (CA) in mice
Source: PLoS One. 2018 Jul 30;13(7):e0201203. doi: 10.1371/journal.pone.0201203 (PMC6066223; doi:10.1371/journal.pone.0201203)
Supplement: S2 Table — (PDF) [file pone.0201203.s003.pdf]

S2 table . Component of basal diet (MF®†, in 100g)

Moisture(g)	7.9
protein(g)	23.1
lipid(g)	5.1
ash (g)	5.8
fiber (g)	2.8
Soluble nitrogen-free product (g)	55.3
Calories (kcal)	359
**Vitamins**	
Vitamin A * (IU)	1283
Vitamin D 3 (IU)	137
Vitamin E (mg)	9.1
Vitamin K 3 ** (mg)	0.04
Vitamin B 1 (mg)	2.05
Vitamin B 2 (mg)	1.1
Vitamin C (mg)	Four
Vitamin B 6 (mg)	0.87
Vitamin B 12 (μ g)	5.5
Inositol (mg)	439
Biotin (μ g)	27
Pantothenic acid (mg)	2.45
Niacin (mg)	10.61
Choline (g)	0.18
Folic acid (mg)	0.17
**Minerals**	
Calcium (g)	1.07
Phosphorus (g)	0.83
Magnesium (g)	0.24
Sodium (g)	0.19
Potassium (g)	0.9
Iron (mg)	10.6
Aluminum (mg)	2.1
Copper (mg)	0.78
Zinc (mg)	4.89
Cobalt (mg)	0.1
Manganese (mg)	4.84
Ca / P	1.29
Ca / Mg	4.5
K / Na	4.77

†; Oriental Yeast Co., Ltd., Tokyo, Japan.
